# The relationship between healthcare workers’ perceptions of epidemic management and manager support in a healthcare institution during the COVID-19 pandemic: scale development study

**DOI:** 10.3389/fpubh.2025.1477961

**Published:** 2025-07-15

**Authors:** Hafize Boyacı, Selma Söyük

**Affiliations:** ^1^Fikret Biyal Central Research Laboratory, Cerrahpaşa Medical Faculty, İstanbul Üniversity-Cerrahpaşa, Istanbul, Türkiye; ^2^Department of Health Management, Faculty of Health Sciences, Istanbul University-Cerrahpasa, Istanbul, Türkiye

**Keywords:** epidemic management, manager support, scale development, pandemic, structure equality model, correlation

## Abstract

**Introduction:**

The aim of the study is to determine the relationship between epidemic management and manager support in healthcare institutions during the COVID-19 pandemic through the perception of healthcare workers. In the research, a scale development study was conducted for the “Epidemic Management Perception Scale”. This scale determines the epidemic management practices in health institutions through the perceptions of healthcare professionals during the COVID-19 pandemic.

**Methods:**

The study is a methodological quantitative research. The data were collected from 365 healthcare workers using a systematic random sampling method. Exploratory factor analysis and confirmatory factor analysis were performed for the validity of the “Epidemic Management Perception” Scale. Cronbach’s Alpha value was used in the reliability analysis of the scale. The theoretical model was tested using the Structural Equation Model.

**Results:**

Epidemic Management Perception Scale was introduced to the literature as a valid and reliable assessment form. In the research, relationships between variables were modeled using structural equation modeling. As a result of the research, a high positive and significant relationship was found between healthcare workers’ perception of epidemic management and manager support in the healthcare institution during the COVID-19 pandemic (*r* = 0.606; *R*^2^: %36). In addition, a highly positive and significant relationship was found between healthcare workers’ perception of manager support and epidemic management sub-dimensions. (Planning *R*^2^: 30.8%, Organization *R*^2^: 26. 2%, Management *R*^2^: 21.9% and Control *R*^2^: 36.2%).

**Discussion:**

It was determined that manager support played an important role in healthcare workers’ positive response to epidemic management practices in healthcare institutions. The supportive attitude of managers in health institutions is the most important factor in overcoming the epidemic process. It is important to inform health managers about the importance of manager support and to organize the necessary training activities.

## 1 Introduction

Throughout history, humanity has been confronted with numerous epidemic diseases. The identification and detection of such epidemics have traditionally required significant time. In particular, the emergence of more complex and interdependent social structures has facilitated the spread of infectious diseases. However, knowledge regarding the origins of epidemics and their modes of transmission has increased over time. Several epidemics have resulted in significant mortality rates and widespread societal disruption. A variety of infectious diseases—including acute hemorrhagic conjunctivitis (AHC), acquired immunodeficiency syndrome (AIDS), cholera, dengue fever, influenza, plague, severe acute respiratory syndrome (SARS), scabies, and West Nile virus—have spread among populations over time. One key characteristic of epidemic diseases is that they affect not only the infected individuals but also society as a whole. With globalization, the threat of epidemics has increased, prompting national and international cooperation and communication aimed at providing detailed information about pathogens, preventing their spread, and taking proactive measures against potential threats. In light of the experiences gained, the development of methods for investigating and treating diseases, taking preventive measures, and raising public awareness are among the positive impacts of globalization.

## 2 A literature review

### 2.1 Epidemic management in healthcare institutions during the COVID-19 pandemic

The coronavirus outbreak first emerged in China and subsequently spread across the globe. The fact that the pandemic led to the deaths of millions of people demonstrated that it constitutes a global public health issue. The World Health Organization (WHO) declared it a pandemic on March 11, 2020. Following this declaration, disease control measures were implemented, and efforts to halt the spread of the virus commenced.

The coronavirus epidemic had its most significant impact on the healthcare sector. The COVID-19 outbreak can be considered a crisis, and the management of the outbreak can be defined as a process. It is essential to determine management strategies during the pandemic and ensure that the process is managed in a controlled manner. This situation has underscored the critical importance of robust health systems. Healthcare institutions must be administratively prepared for epidemics, and contingency plans should be developed to address potential challenges. This is crucial for ensuring the continuity of healthcare services (1). In response to epidemics, healthcare institutions should adopt diverse strategies that enable rapid and effective decision-making (2).

This situation has underscored the critical importance of robust health systems. Healthcare institutions must be administratively prepared for epidemics, and contingency plans should be developed to address potential challenges. This is crucial for ensuring the continuity of healthcare services (1). In response to epidemics, healthcare institutions should adopt diverse strategies that enable rapid and effective decision-making (2).

In order to be adequately prepared for an epidemic, it is essential to develop comprehensive action plans, approach the situation with the necessary seriousness, and strategically plan the allocation of resources required during the epidemic period (1). The management of healthcare institutions is inherently complex due to their multidisciplinary structure (3). Consequently, decision-makers must identify and implement a range of strategies to effectively manage the epidemic process (4). In addition to the adverse effects of epidemics on public health, significant social, economic, and administrative consequences also emerge. Lessons learned from previous epidemics offer valuable insights that can help mitigate the negative impacts of future pandemics (5). The *COVID-19 Strategic Preparedness and Response Plan* outlined key priority areas and methodologies for combating the pandemic. These include preventive measures to halt the spread of the virus, stabilization of patients, restructuring of the healthcare system, development of preparedness and response strategies to counter the epidemic’s negative effects, efficient and effective use of resources, and ensuring the safety of healthcare personnel (6, 7).

In order to ensure the uninterrupted provision of healthcare services, it is essential to establish effective epidemic management practices. During the epidemic process, it was necessary to regulate issues such as excessive workload, labor loss, physical conditions, and methods of protecting employees from the effects of the virus and training activities. The epidemic process must be approached as a crisis, with a clearly defined management strategy, the development of strategic plans, and effective control mechanisms. The implementation and periodic revision of training programs are essential to ensure the safety of healthcare workers (8). Continuous monitoring of patients is necessary, and data regarding case increases should be systematically collected and analyzed (9). Workforce requirements resulting from increased workload, along with the monitoring and management of medical supplies and equipment, must be planned in alignment with evolving conditions (10). During an epidemic, it is essential to define management strategies to ensure that the process is handled in a controlled manner. Situations that arise suddenly and unexpectedly are referred to as crises. Crisis management is defined as the effort to protect an organization from the potential adverse effects of unexpected events through the evaluation and implementation of crisis management activities by administrators (1). The activities carried out from the beginning to the end of a crisis constitute the crisis management process. In crisis situations, it is necessary to identify the source of the crisis, determine preparatory measures, assess the process, implement preventive actions, and identify and correct potential negative impacts of the crisis (11).

In many national and international studies, scale development studies on epidemic and crisis management have been conducted, and the process has been evaluated. Penrose (12) stated in her study that the company’s crisis perception has a primary impact on crisis management activities and that threats and opportunities should be examined when planning the crisis management activities. Abraham and Schaubroeck (13) developed a scale to determine the current situation in terms of emergency and crisis preparedness and the preparedness to cope with future crises. As a result of the research conducted by Nevala and Vuorela (14) with a semi-structured questionnaire, it was observed that infection prevention controls were at a critical level. The need to organize the motivation of employees in a way that is compatible with the crisis and the importance of primary healthcare services were emphasized. Mishra et al. (15) proposed a new health system model to improve health services for the society. The model is a three-based health model under the titles of patient care and management, public health management (hygiene, etc.), and health technology. He argued that it would be more effective to turn models with technological infrastructure into a policy to combat COVID-19. He suggested that early diagnosis and treatment methods would be effective thanks to advanced technology. Nano and digital technology and materials science are some of them (15). Chatzittofis et al. (16) emphasized in their research that stress and organizational support should be evaluated together in order to strengthen healthcare professionals during the pandemic (16). In the study by Dehnavieh and Kalavani (17), the importance of employee approval and appreciation, ensuring work-life balance, and regulating working and resting hours is mentioned. In addition, protecting employees from external influences with the support of colleagues and paying attention to risk factors within the organization are also important issues. The importance of teamwork, employee control, and participation in work is also mentioned (17). In a study, international and national organizations emphasized the importance of acting with a common perspective in the decisions and policies implemented in the fight against COVID-19 (18). Some of the scale development studies related to crisis/epidemic in the literature are presented in the Supplementary material 1.

Unlike studies in the literature, our research was evaluated from the perspective of health professionals who experienced the epidemic management practices process firsthand. Therefore, the developed scale is important because it evaluates the situation from a realistic perspective. The Epidemic Management Perception (EMP) scale will be an important guide for the literature in terms of determining the attitudes and behaviors of healthcare professionals toward the epidemic management practices of healthcare institutions. The scale measures the perception of comprehensive epidemic management based on the theory of management functions.

### 2.2 Manager support in healthcare institutions during the COVID-19 pandemic

During the COVID-19 pandemic, organizations have gone through a difficult period while trying to maintain their existence and adapt to the new situation. In this process, employees and managers have played an important role. Studies show that managers make efforts to help their employees adapt to the working environment and changing conditions in crisis and epidemic situations (19). During the pandemic, managers tried to achieve the goals and objectives of the organization while also working to provide the necessary conditions and employee coordination for the new situation. Managers had to struggle with factors, such as burnout, bad habits, process management, and loss of motivation, that employees face in difficult conditions. During the pandemic, organizational managers, like employees, tried to fulfill their duties under risk (20). In the past, it was necessary to prioritize management activities in epidemics, natural disasters, and crises. According to past experiences, ensuring correct and effective communication is important for the continuity of trust in the employees of institutions. The incomplete and inaccurate information shared with employees and the public increases fear and anxiety. Communication has also played an important role in the COVID-19 pandemic. In order to create public awareness about the transmission, spread and treatment of the disease, it is important for authorized persons to receive accurate information. Properly informing healthcare professionals is essential for reducing anxiety, stress, and emotional uncertainty and for the continuity of service. The information shared with employees during the outbreak management process ensures the trust that employees have in their managers (21). Health institution managers, employees and the entire society have the same concerns during the epidemic process. Therefore, it is important to prepare the necessary measures and plans comprehensively in the fight against the epidemic (22). Healthcare workers all over the world have struggled with risking their own health during the pandemic. Healthcare managers are an effective tool in improving adverse conditions and increasing motivation with the support they show to their employees. A positive and supportive relationship between employees and managers is important for the process to go through easily. The way to increase trust in managers is to support employees, take protective measures against the stress and anxiety caused by the pandemic, and ensure that employees participate in decisions (21). Managers who value their employees and have strong communication with them have contributed to the organization achieving its goals, especially in the difficult working conditions experienced during the COVID-19 pandemic. Research shows that there is an inverse relationship between the increased perception of managerial support and the intention to leave the job (23).

Healthcare workers and healthcare managers have taken on a lot of responsibility during the COVID-19 pandemic. The research attempted to determine healthcare workers’ perceptions of epidemic management procedures in healthcare institutions. At the same time, an attempt was made to emphasize the importance of the support healthcare workers receive from their managers during a difficult process such as the pandemic.

The aim of the research was to reveal how healthcare institution workers perceived the relationship between epidemic management and manager support during the COVID-19 pandemic. For this reason, a comprehensive and up-to-date “Epidemic Management Perception” (EMP) Scale was developed and introduced into the literature.

In the research, a model was created using structural equation modeling (SEM) to determine the relationships between healthcare workers’ perceptions of Epidemic Management and manager support during the pandemic period. The research reveals the attitudes and thoughts of healthcare workers toward the epidemic management implemented in healthcare institutions during the COVID-19 pandemic. At the same time, a study that clearly evaluates the importance of manager support has not been conducted and is thought to be the first.

The “Materials and Methods” section of the study provides information about the general structure of the study, its purpose, importance and data collection methods, scale development, validity and reliability steps. In the Findings section, the hypothesis and the research model were tested using the structural equation model. In the “Discussion” section, the research findings were compared with international and national publications. The results and recommendations reached in the study were presented.

The research data were obtained by conducting a survey of healthcare professionals working at a university hospital in Istanbul. The findings were used to determine how healthcare workers perceived epidemic management practices in healthcare institutions during the pandemic. Experiences gained from the COVID-19 pandemic are important in terms of being prepared for future pandemics. The research, which determines the perceptions of healthcare workers who experienced the pandemic process intensively regarding process management, is of great importance in terms of the literature. The importance of the support healthcare workers receive from their managers during the pandemic process was revealed. The support that healthcare workers receive from their managers has a significant impact on their adaptation to the epidemic management process and their ability to overcome the process more easily.

In this context, the research model was tested by determining the relationships between epidemic management and perception of manager support.

## 3 Materials and methods

The research is methodological quantitative research. The population of the study consists of healthcare professionals working in different titles at a university hospital in Istanbul, Turkey. The study was conducted between March and October 2023. The research was conducted with the Ethics committee permission numbered 2022/106 (numbered E-74555795-050.01.04-567445; 16.12.2022) and institutional permission numbered 512477 (18.10.2022) were obtained. The research was conducted, and the necessary consent forms for the survey study were obtained. This study was performed in accordance with the guidelines of the Declaration of Helsinki and was approved. Participants answered the survey voluntarily.

### 3.1 Purpose of the research

In order to be prepared for future epidemics, it is important to determine the perception of healthcare professionals regarding epidemic management in healthcare institutions. In the research, the “EMP” scale development study was conducted to determine how management activities work in the fight against the COVID-19 pandemic, how they should be and their adequacy. The “EMP” Scale aims to determine, monitor, and measure epidemic management implemented in healthcare institutions and to guide the development of epidemic management skills in future epidemics and disasters.

The other purpose of the research is to create a model using SEM to determine the relationship between healthcare workers’ perceptions of epidemic management and manager support during the pandemic.

### 3.2 Population and sample

The universe of the study consists of healthcare professionals working in different titles at a university hospital in Istanbul. The systematic random sampling method, which is a probability sampling method, was used in the study. A sample selection method was used since it is difficult and costly to reach the entire universe. The systematic random sampling method is used when the universe is large, which increases the representativeness of the study. The systematic random sampling method was used to prevent bias during data collection. The universe was represented more strongly using the listing and numbering method (24). The list of people constituting the universe in the study (*N* = 3,148) was made, and the sample interval (k) was determined with the *N*/*n* formula. A random starting point between 1 and the sample interval was selected using the Excel method. In the study, power analysis was also performed to test sample adequacy. As a result of the power analysis performed with the G * Power application within the scope of the research, it was determined that the sample size of 262 was sufficient [*t*-test: effect size *f*: 0.2, alpha (*α*): 0.05, power (1-*β*): 0.95: actual power: 0.950; *F*-test: effect size *f*: 0.3, alpha (α): 0.05, power (1-β): 0.95: actual power: 0.950]. The research was conducted with 365 healthcare workers. The people to be sampled were selected by starting from the determined person and adding them to the sample interval. In order to avoid missing data and underfilling errors in the study, a total of 365 people were reached, including 151 doctors, 117 nurses, and 97 other healthcare personnel. Data were collected using the survey method in the study.

### 3.3 Data collection tool

The research data were collected using the survey method. The survey form consisted of three sections: demographic information form, EMP (Epidemic Managenet Perception) Scale form, and MSP (Manager Support Perception) Scale form.

#### 3.3.1 Epidemic management perception scale

A study was conducted to develop the EMP Scale. The stages of developing the “Epidemic Management Perception” Scale are as follows:

*Epidemic management perception scale development stages*: as a result of the literature review, since a comprehensive scale suitable for the research topic on the perception of epidemic management could not be found, the scale development method was decided. The steps in the analysis of developing a valid scale in the research are surface, content, and construct validity. In the EMP Scale validity test steps; creating the item pool in the surface validity stage, presenting the item pool to expert opinion in the content validity stage, pre-test and sampling application steps in the construct validity stage were followed.

*Epidemic management perception scale surface validity*: in the scale development study, deductive, inductive or both item generation methods can be used (25). The deductive method was used in the study. The item pool of the EMP Scale was created in line with the literature to ensure surface validity.

*Epidemic management perception scale item pool*: the purpose of creating an item pool is to determine the conceptual framework and the statements that can be included in the scale. The deductive method used in the item pool creation phase of the scale development study is based on a literature review on the subject. If there is sufficient theoretical information in the literature, this method is preferred. The item pool of the scale was created with 56 items after a comprehensive literature review (12, 14–17, 26–44) (Supplementary Table S1), and consultation with three health managers and health workers in the health institution. The 56-item scale was reduced to 40 items by eliminating expressions with the same meaning. The 5-point Likert-type rating measurement method was used in the EMP Scale. The answers given to the scale statements were graded as Strongly Disagree (1), Disagree (2), Undecided (3), Agree (4), and Strongly Agree (5). The items of the scale to be used in scale development consist of closed-ended questions.

*Epidemic management perception scale content validity*: it is a type of validity in which expert opinions are taken to check the suitability of scale expressions. Lawshe’s (45) technique was used when creating an “Expert Opinion Form” in the research. According to the Lawshe technique, 5–40 expert opinions can be applied. The opinions of eight experts were consulted in the research. The experts consist of six academicians and two health managers. In the content validity phase, 7, 24, 26, and 37 items are removed from the scale. Item 39 has been changed. The content validity rate (KGI = 0.91) for a total of 36 scale expressions. The scale items were finalized and made ready for pre-test.

#### 3.3.2 Manager support perception scale

The “Manager Support Perception” scale, developed by McGilton (46) and adapted into Turkish by Boyacı and Söyük (47), was used. The scale was developed to measure the abilities of managers regarding the support they show to their employees. It is a suitable tool for evaluating the perception of manager support of healthcare workers during the pandemic period. The scale developed by McGilton (46) is used by adapting it to foreign languages. Rodríguez-Monforte et al. (48) and Um-e-Rubbab et al. (49) used this scale in their studies. Cronbach’s alpha of the original scale takes values between 0.85 and 0.97. Tian et al. (50) adapted the original scale to Chinese in their study and found the Cronbach’s alpha value to be 0.85. Boyacı and Söyük (47) found the Cronbach’s alpha value of the 15-item, single-dimensional Managerial Support Perception Scale to be 0.973. The scale is graded at 5 points. The answers are Never (1), Seldom (2), Occasionally (3), Often (4), and Always (5), and the answers give an overall score between a minimum of 15 and a maximum of 75.

### 3.4 The theoretical model

**Figure 1:**
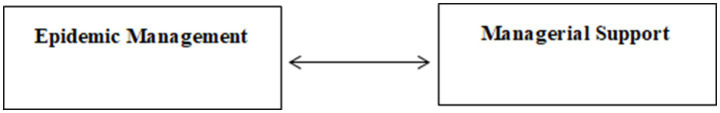
The theoretical model.

The research was planned to determine the relationship between epidemic management and managerial support implemented in healthcare institutions during the epidemic process, through the perception of healthcare workers. In the research, the relationship between epidemic management and manager support was tested using the structural equation model. The theoretical model that forms the basis of the research is presented in Figure 1. The relationships between the variables were revealed. Statistical analysis of the obtained data was performed, the research hypothesis was tested, and the findings were explained.

### 3.5 Statistical analysis

Statistical analyses were conducted using IBM SPSS (v22), LISREL 8.8, and MS Excel 16. Descriptive statistics were calculated for participant characteristics, and the EMP Scale’s validity and reliability were assessed through KMO, Bartlett’s test, Cronbach’s alpha, and the 27% discrimination index. Normality was tested using skewness, kurtosis, and the Kolmogorov–Smirnov test. The scale’s structure was examined using exploratory factor analysis (EFA) and confirmed through confirmatory factor analysis (CFA). Pearson correlation was used to assess the relationship between EMP and MSP. A theoretical model was developed and tested using SEM, with epidemic management as the independent variable and managerial support as the dependent variable. Model fit indices were evaluated, and all analyses were conducted with a 5% margin of error and a 95% confidence interval (*p* < 0.05).

In the study, the data were analyzed and evaluated in the following four steps: preparation before data analysis, descriptive statistical evaluation of the obtained data, testing the model with the data using the SEM, evaluating the fit indices, and testing the research hypothesis.

### 3.6 Hypothesis

*H0:* There is no significant relationship between healthcare workers’ perceptions of epidemic management and manager support during the COVID-19 pandemic.

*H1:* There is a significant relationship between healthcare workers’ perceptions of epidemic management and manager support during the COVID-19 pandemic.

## 4 Results

### 4.1 Epidemic management perception scale construct validity

#### 4.1.1 Evaluation by pre-test

A pre-test was applied to 50 healthcare workers (*n* = 50). In the statistical analysis phase of the data obtained as a result of the pre-test application, EFA, normality tests, and item analysis were applied. Pilot application result: Chi-square value = 1286.362; df = 465; KMO = 0.794; Bartlett test = 0.000 was found. In the EFA analysis, principal components and direct oblimin rotation methods were applied to the data and a 5-factor structure was obtained. EMP Scale pre-test Cronbach’s alpha coefficient = 0.951. The scale mean was found to be 2.93 ± 0.602. As a result of the pre-test application, it was determined that the scale met the validity and reliability conditions. The scale was finalized, and the application phase was started on the target audience (*n* = 365). The data were analyzed with the SPSS 22.0 package program.

#### 4.1.2 Evaluation by target population

It was applied to the target population with 365 healthcare workers (*n* = 365). As a result of the statistical analysis of the data obtained as a result of the sampling application, KMO = 0.95, Bartlett test *p* < 0.001. The results show that the data are suitable for EFA. In the correlation matrix table, the correlation coefficient between all items is >0.30. According to the results obtained, no items were removed from the scale (51). It was concluded that the dataset is suitable for factor analysis.

Principal component analysis and the direct oblimin rotation method were used in EFA analysis. Initially, a 5-factor structure was obtained. Items with overlapping and factor load below 0.1 were excluded from the scale (7, 10, 15, 21). In the analysis, rotation allowed up to 14 processing and the 4-factor structure was completed by performing four repetitions. The lowest factor load was 0.497, and the highest factor load was 0.905. The item analysis of the EMP Scale is given in Table 1, while the factor distribution and factor loading are given in Table 2.

**Table 1:** Epidemic management perception scale item analysis.

Items	*n*	Average	SS	Factor load	Item-total correlation	Squared multiple correlations
EMP1	365	2.62	0.975	0.516	0.527	0.598
EMP2	365	2.78	0.978	0.756	0.727	0.763
EMP3	365	2.61	1.093	0.623	0.665	0.562
EMP4	365	2.93	1.039	0.684	0.746	0.673
EMP5	365	2.91	0.907	0.587	0.657	0.577
EMP6	365	3.27	0.944	0.600	0.653	0.588
EMP7	365	3.08	0.987	0.678	0.747	0.698
EMP8	365	2.95	0.986	0.693	0.744	0.672
EMP9	365	2.35	0.884	0.431	0.598	0.459
EMP10	365	2.57	1.037	0.670	0.652	0.629
EMP11	365	2.99	1.074	0.471	0.623	0.500
EMP12	365	2.45	1.035	0.660	0.715	0.646
EMP13	365	2.52	1.065	0.674	0.733	0.662
EMP14	365	2.55	1.022	0.649	0.721	0.657
EMP15	365	2.66	1.016	0.790	0.797	0.773
EMP16	365	2.26	0.994	0.795	0.740	0.768
EMP17	365	2.15	1.063	0.643	0.621	0.652
EMP18	365	2.87	1.043	0.756	0.646	0.727
EMP19	365	2.90	1.040	0.857	0.701	0.791
EMP20	365	2.86	1.004	0.804	0.706	0.722
EMP21	365	2.76	1.011	0.732	0.742	0.692
EMP22	365	3.13	0.970	0.733	0.763	0.730
EMP23	365	3.55	0.992	0.444	0.468	0.312
EMP24	365	3.19	0.975	0.721	0.757	0.716
EMP25	365	3.04	1.036	0.738	0.760	0.739
EMP26	365	3.42	0.971	0.746	0.656	0.640
EMP27	365	3.02	0.987	0.731	0.769	0.753

EMP, Epidemic Management Perception scale items; SS, standard deviation.

**Table 2:** Epidemic management perception scale factor loadings.

Items	Factor 1	Factor 2	Factor 3	Factor 4
EMP1	**0.728**	0.114	0.097	−0.127
EMP2	**0.758**	0.144	0.170	0.132
EMP3	**0.698**	0.083	−0.192	−0.127
EMP4	**0.657**	0.093	−0.209	−0.003
EMP5	**0.687**	−0.005	−0.177	−0.007
EMP6	**0.652**	−0.043	0.051	0.278
EMP7	**0.632**	0.016	−0.101	0.218
EMP8	**0.696**	0.044	−0.097	0.111
EMP9	0.093	**0.497**	0.009	0.163
EMP10	0.025	**0.819**	0.209	0.112
EMP11	0.086	**0.481**	0.041	0.255
EMP12	0.089	**0.731**	−0.045	0.000
EMP13	−0.022	**0.714**	−0.158	0.069
EMP14	0.045	**0.703**	−0.100	0.042
EMP15	0.022	**0.785**	−0.146	0.026
EMP16	0.012	**0.905**	−0.039	−0.083
EMP17	−0.031	**0.863**	−0.038	−0.135
EMP18	−0.091	0.166	**−0.726**	0.178
EMP19	0.030	0.138	**−0.801**	0.079
EMP20	0.245	−0.011	**−0.739**	0.074
EMP21	0.244	0.113	**−0.597**	0.098
EMP22	0.141	0.128	−0.198	**0.582**
EMP23	−0.081	0.008	−0.174	**0.609**
EMP24	0.262	0.155	0.007	**0.586**
EMP25	0.313	0.067	−0.042	**0.594**
EMP26	−0.046	0.129	−0.039	**0.799**
EMP27	0.358	0.048	−0.081	**0.542**

Extraction method: principal component analysis. Rotation method: Oblimin with Kaiser normalization. Rotation converged in 14 iterations. EMP, Epidemic Management Perception scale items. Significant at the Factor load >0.3.

The 4-factor structure with an eigenvalue >1 explains 67.34% of the total variance. Total Eigenvalues explains factors: Factor 1: 13.999, Factor 2: 1.652, Factor 3: 1.464, and Factor 4: 1.069. The 4-factor structure with an eigenvalue >1 explains 67.34% of the total variance. The mean and standard deviation scores of the EMP Scale were determined as 2.82 ± 0.72. The mean and standard deviation of the EMP Scale sub-dimensions were determined as follows: P: 2.89 ± 0.77, S: 2.49 ± 0.80, M: 2.84 ± 0.90, and C: 3.22 ± 0.80. Cronbach’s alpha coefficient: Factor 1: 0.911, Factor 2: 0.924, Factor 3: 0.91, and Factor 4: 0.901.

#### 4.1.3 Tests of reliability

The general scale item reliability was found as Cronbach’s alpha = 0.963. It was determined that the reliability of the general and sub-factors of the scale was quite high. In another reliability analysis of the EMP Scale, the significance test of the difference between groups was used as a 27% method. A statistically significant difference was found between the mean scores of the upper group and the lower group of the scale. This showed that the discriminatory power of the scale was high (*p* < 0.05) (Levene’s *F* = 4.792, *p* < 0.03). It was concluded that the reliability of the general scale and its factors was high.

#### 4.1.4 Epidemic management perception scale confirmatory factor analysis

**Figure 2:**
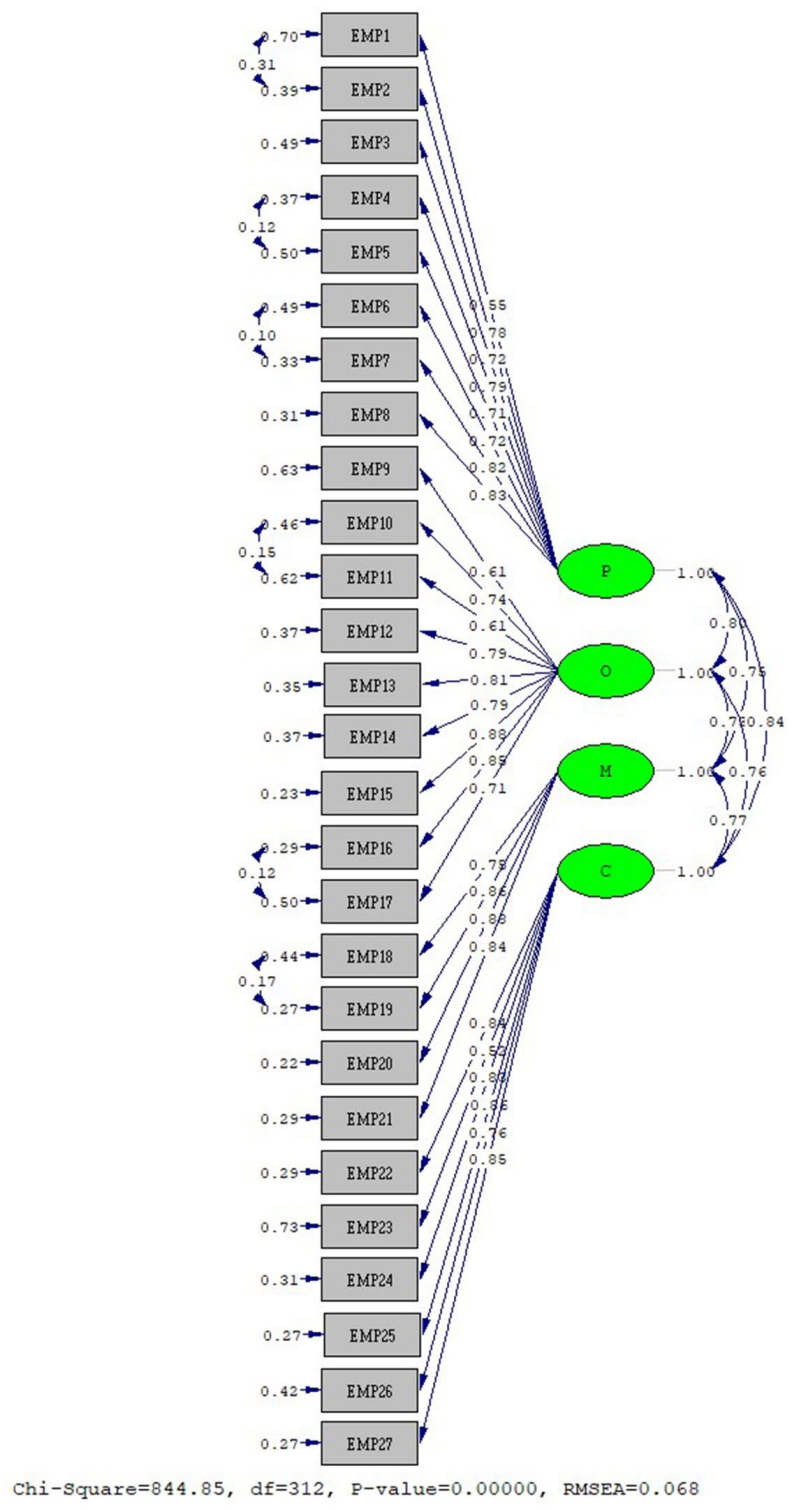
EMP scale first-level CFA standardized solution graph.

**Figure 3:**
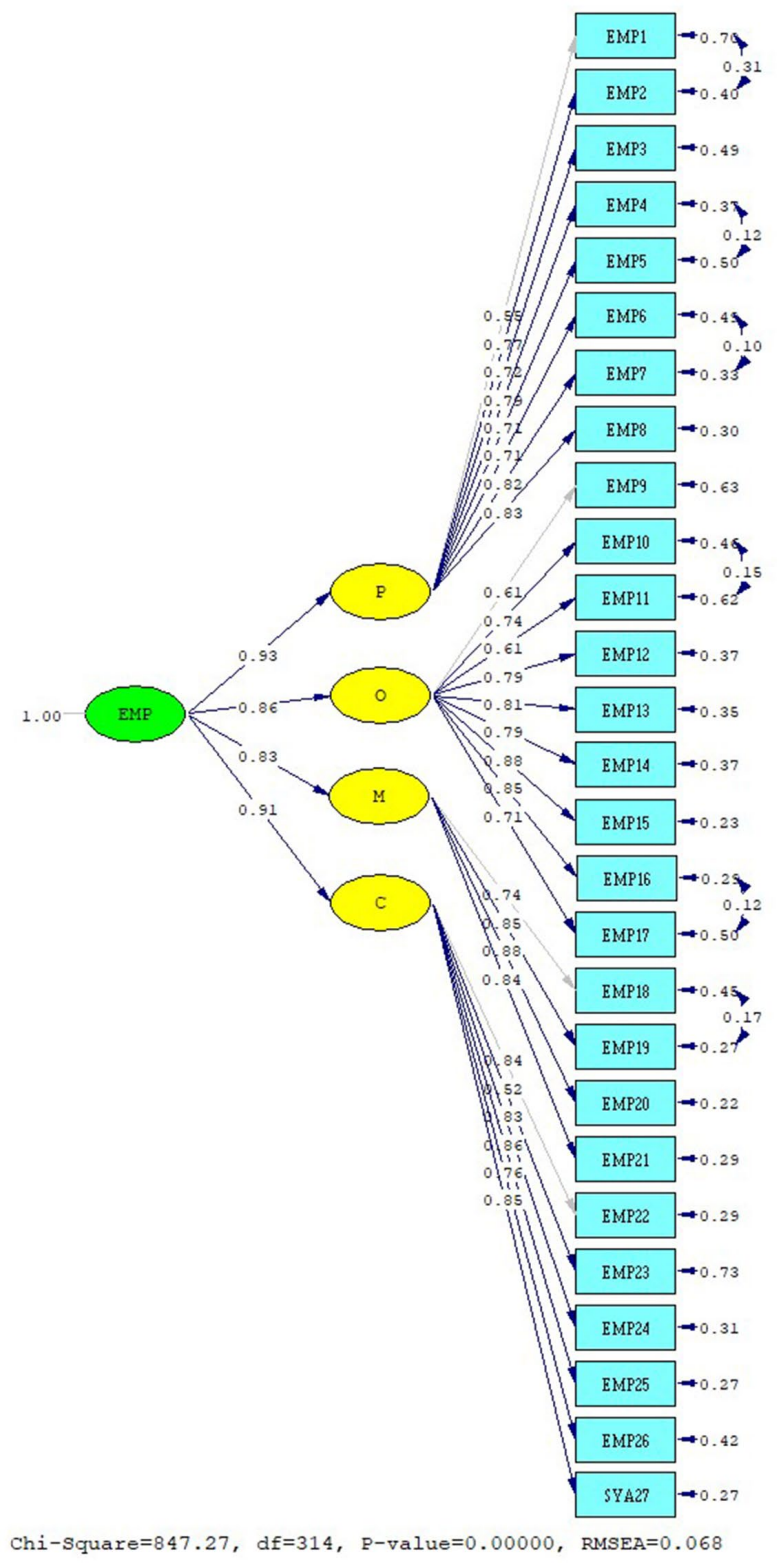
EMP scale second-level CFA standardized solution graph.

In scale development, the construct validity of the data obtained from EFA is confirmed by CFA (52). The construct validity of the EMP Scale was provided with CFA analysis. As a result of CFA, a modification process was applied to reach the fit values. The analysis was repeated by applying modifications to the items EMP1-2, EMP4-5, EMP6-7, EMP10-11, EMP16-17, and EMP18-19. The fit values of the first-level model are as follows: RMSEA: 0.068, Chi-square/SD (CMIN/DF): 2.76, CFI: 0.98, GFI: 0.85, AGFI: 0.82, NFI: 0.97, sRMR: 0.43, RMR: 0.044, RFI: 0.97, and IFI: 0.98. And standardized values were reached. The first-degree standardized solution graph of the “EMP” Scale CFA is given in Figure 2. The fit values of the second-level model are as follows: RMSEA: 0.068, Chi-square/SD (CMIN/DF): 2.76, CFI: 0.98, GFI: 0.85, AGFI: 0.82, NFI: 0.97, IFI: 0.98, NNFI: 0.98, sRMR: 0.044, RMR: 0.045, RFI: 0.97, and Critical N (CN) = 158.22. As a result of the second-degree CFA of the EMP Scale, a structurally valid model was obtained (53). The second-degree standardized solution graph of the EMP Scale is given in Figure 3. EMP Scale average variance extracted (AVE): 0.596, composite reliability (CR): 0.975 was found. The item statistics regarding the CFA findings of the EMP Scale are given in Table 3.

**Table 3:** Epidemic management perception scale CFA results of study measures.

Item	Λ	R^2^	Error variance	*t*	*p*	AVE	CR
						0.596	0.975
EMP1	0.55	0.30	0.70	10.94	0.051		
EMP2	0.78	0.61	0.39	17.31	0.032		
EMP3	0.72	0.51	0.49	15.36	0.047		
EMP4	0.79	0.63	0.37	17.72	0.035		
EMP5	0.71	0.50	0.50	15.04	0.034		
EMP6	0.72	0.51	0.49	15.30	0.036		
EMP7	0.82	0.67	0.33	18.53	0.029		
EMP8	0.83	0.69	0.31	19.18	0.027		
EMP9	0.61	0.37	0.63	12.60	0.038		
EMP10	0.74	0.54	0.46	16.06	0.040		
EMP11	0.61	0.38	0.62	12.66	0.056		
EMP12	0.79	0.63	0.37	17.90	0.033		
EMP13	0.81	0.65	0.35	18.43	0.033		
EMP14	0.79	0.63	0.37	17.87	0.032		
EMP15	0.88	0.77	0.23	21.04	0.022		
EMP16	0.85	0.71	0.29	19.73	0.025		
EMP17	0.71	0.50	0.50	15.20	0.045		
EMP18	0.75	0.56	0.44	16.07	0.042		
EMP19	0.86	0.73	0.27	19.85	0.049		
EMP20	0.88	0.78	0.22	20.76	0.026		
EMP21	0.84	0.71	0.29	19.35	0.029		
EMP22	0.84	0.71	0.29	19.68	0.024		
EMP23	0.50	0.27	0.73	10.42	0.054		
EMP24	0.83	0.69	0.31	19.14	0.026		
EMP25	0.86	0.73	0.27	20.15	0.026		
EMP26	0.76	0.58	0.42	16.80	0.033		
EMP27	0.85	0.73	0.27	19.96	0.024		

EMP, Epidemic Management Perception scale items; AVE, average variance extracted; CR, composite reliability (CR); Λ, factor load.

In the evaluation of the CFA analysis, the fit between the model and theory is decided according to the fit index values available in the literature. Various fit values are used in the evaluation phase of the theoretical model and the data. Even if the factor loadings of the model are good, the decision is made according to the achievement of fit indices. Although there are many different fit indices in the literature, there is no consensus on which one to accept (54). CFA analysis fit index values are examined, and model fit is decided in scale validity and reliability studies. The literature states that some or all of the fit values can be used. In the study, Chi-square/SD (CMIN/DF), RMSEA, CFI, sRMR, NFI, RFI, RMR, IFI, NNFI, relative GFI, and AGFI values were used. *χ*^2^ value: Chi-square value is expected to be insignificant. Examines the fit of the universe covariance matrix and the sample covariance matrix. It means that there is a difference between the matrices. The test result is expected to be insignificant (54). *χ*^2^/SD value: Instead of *χ*^2^, the *χ*^2^/SD ratio, which is less affected by the sample, is used. It is obtained by dividing the *χ*^2^ value by the degree of freedom. It should be 2 or less. A total of 5 or less is an acceptable value (54). EMP Scale Chi-square/SD (CMIN/DF): The value 2.76 is within acceptable limits. Root-mean-square error of approximation (RMSEA): It takes values between 0 and 1 (51, 54). It is desired to give values close to “0” (minimum error between latent and observed matrices). Values equal to or less than 0.05 indicate a perfect fit, and values up to 0.08 indicate an acceptable. EMP Scale RMSEA: 0.068 meets the expectation of minimum error between the latent and observed matrices. It was found within acceptable limits. Comparative Fit Index (CFI): It gives the difference between the model established by assuming that there is no relationship between the variables and the null model. It is a criterion that takes into account the degree of freedom in the model and the sample size in the evaluation of model fit. Its value varies between 0 and 1 (51, 54). A CFI value above 0.95 indicates a perfect fit, and a value above 0.90 indicates a sufficient fit. EMP Scale CFI = 0.98 was determined. It shows a perfect fit. Goodness-of-Fit Index (GFI): It shows how well the model measures the Covariance matrix in the sample (51, 54). The GFI value varies between 0 and 1. If the GFI is above 0.90, it is a good model indicator. Adjustment Goodness-of-Fit Index (AGFI): it is the GFI value adjusted by taking into account the sample size. A total of 0.90 and above are considered a good fit. It takes values between 0 and 1 (51, 54–56). EMP Scale was determined as GFI: 0.85, AGFI: 0.82. Root-mean-square residual (RMR): as the value approaches zero, it is understood that the tested model shows a better fit. Standardized root-mean-square residual (sRMR): Its standardized form is called the sRMR fit index (51). sRMR: 0.044, RMR: 0.045 values indicate that the model’s data fit is within acceptable limits (54, 55). As a result of the second-level CFA analysis of the EMP Scale, a structurally valid model was obtained. EMP Scale second-level CFA fit values are presented in Table 4. The results show that the model data fit is achieved, and the scale is a valid scale.

**Table 4:** Epidemic management perception scale second-level CFA fit values.

Fit value		Good fit	Acceptable fit
*χ*^2^/df	2.76	0 ≤ *χ*^2^/SD ≤ 2	2 ≤ *χ*^2^/SD ≤ 5
RMSEA	0.068	0.00 ≤ RMSEA ≤ 0.05	0.05 ≤ RMSEA ≤ 0.08
CFI	0.98	0.95 ≤ CFI ≤ 1.00	0.90 ≤ CFI ≤ 0.95
GFI	0.85	0.95 ≤ GFI ≤ 1.00	0.90 ≤ GFI ≤ 95
AGFI	0.82	0.90 ≤ AGFI ≤ 1.00	0.85 ≤ AGFI ≤ 0.90
sRMR	0.044	0.00 ≤ SRMR ≤ 0.05	05 ≤ SRMR ≤ 0.08

*χ*^2^/df, Ki kare; RMSEA, root-mean-square error of approximation; sRMR, standardized root-mean-square residual; GFI, goodness-of-fit index; AGFI, adjusted goodness-of-fit index; CFI, comparative fit index (44).

The final version of the scale was given, and the factors were named as follows: first factor “Planning,” second factor “Organization,” third factor “Management,” and fourth factor “Control” (the final version of the scale is given in Supplementary Table S2). The results of the validity and reliability studies conducted in the scale development part of the research are presented with evidence. The EMP Scale has been brought to the literature as a valid and reliable scale.

As a result of the statistical analysis of data obtained from 365 healthcare workers, a valid and reliable scale was developed. Analyses were carried out with 5% error and a 95% confidence level. EMP Scale normality tests; skewness value = −0.135 ± 0.128 and kurtosis values = 0.243 ± 0.255 were used as normality tests of the scale. Skewness and kurtosis values between (±1) are sufficient for normality (51). The Kolmogorov–Smirnov value is *p* > 0.200, and the Shapiro–Wilk value is *p* > 0.097. It was concluded that the scale showed normal distribution.

### 4.2 Correlation analysis of EMP and MSP scales and testing of hypothesis

Demographic characteristics of healthcare workers included in the study are given in Table 5. Pearson correlation analysis was performed to determine the relationship between epidemic management and manager support perception of healthcare workers during the pandemic period. Hypothesis testing was done using SPSS 22.00 and Lisrel Estimated 8.8 programs. Evidence was strengthened by testing the relationship between epidemic management and manager support with the created model. Correlation significance values were used as *r* < 0–0.20 (no relationship-very weak relationship), 0.20–0.39 (weak), 0.40–0.59 (medium), 0.6–0.79 (high), and 0.80–1.0 (very high relationship) (50).

**Table 5:** Demographic characteristics of the participants.

Variable		Frequency	%
Gender	Female	237	64.9
	Male	128	35.1
Educational Status	High school	15	4.1
	Bachelor degree	167	45.8
	Master vs. doctorate degree	183	50.1
Position Title	Doctor	151	41.4
	Nurse	117	32.1
	Health technician	74	20.3
	Other employees	23	6.3
Working time in the profession	0–5 years	54	14.8
	6–10 years	33	9.0
	11–15 years	64	17.5
	16 and over years	214	58.6
Total		365	100

A highly positive, statistically significant relationship was found between healthcare workers’ perceptions of epidemic management and manager support (*p* < 0.01) (r = 0.606; *R*^2^ = 36.7%). In other words, a positive relationship was found between epidemic management’s perception and perception of managerial support. The support that healthcare workers received from their managers during the pandemic process enabled them to develop positive attitudes and behaviors toward the epidemic management practices implemented in healthcare institutions. As healthcare professionals’ perceptions of managerial support increase, their positive thoughts about epidemic management also increase. The correlation results obtained as a result of the research are presented in Table 6.

**Table 6:** Pearson correlation results for variables.

Correlations							
Scales		Scales		EMP Scales sub-dimensions			
		MSP	EMP	P	O	M	C
MSP	Pearson r	1.00					
	P						
EMP	Pearson r	0.606	1.00				
	P	**0.001**					
P	Pearson r	0.555	0.903	1.00			
	P	0.**001**	0.**001**				
O	Pearson r	0.512	0.908	0.742	1.00		
	P	**0.001**	**0.001**	**0.001**			
M	Pearson r	0.469	0.817	0.650	0.659	1.00	
	P	0.**001**	**0.001**	**0.001**	**0.001**		
C	Pearson r	0.602	0.881	0.743	0.701	0.707	1.00
	P	**0.001**	**0.001**	**0.001**	**0.001**	**0.001**	

MSP, Manager support perception; EMP, Epidemic management perception; P, Planning; O, Organization; M, Management; C, Control. Correlation is significant at the *p* < 0.05.

A highly positive and significant relationship was also found between healthcare professionals’ perception of manager support and the sub-dimensions of the EMP Scale (*p* < 0.01). Planning (*r* = 0.555; *R*^2^ = 30.8%), Organization (r = 0.512; *R*^2^ = 26.2%), Management (r = 0.469; *R*^2^ = 21.9%), and Control (r = 0.602; *R*^2^ = 36%). Employees who receive manager support also exhibit a positive attitude toward each sub-dimension of planning, organization, management, and control, which are epidemic management functions. As the participants’ perception of manager support increases, their positive thoughts in every dimension of epidemic management increase. The high relationship between epidemic management and manager support reveals that manager support is very important for healthcare workers to adapt to the process of epidemic management in healthcare institutions.

### 4.3 Data-model fit review (structural equation modeling)

Theoretical model: It evaluates the managerial support and epidemic management perceived by employees during the epidemic process in health institutions. SEM analysis was preferred to test the model with research data. SEM resembles CFA. However, unlike CFA, explanatory relationships between latent variables are also taken into account (57). SEM, a theory-based analysis method, reveals latent (unobservable) variables by making use of observed variables. SEM is used to detect the effects of latent (unobservable) variables and test the theoretical model as a whole by revealing the relationship between them (58). The SEM model analyses the interaction of variables while accounting for measurement errors (59, 60). The multiple models, influenced by variables, provide a holistic perspective. It tests the research question as a whole (61). SEM was preferred in the research to present the perception of healthcare professionals regarding the epidemic process from a holistic perspective (62).

#### 4.3.1 MODEL: relationship between epidemic management and manager support

**Figure 4:**
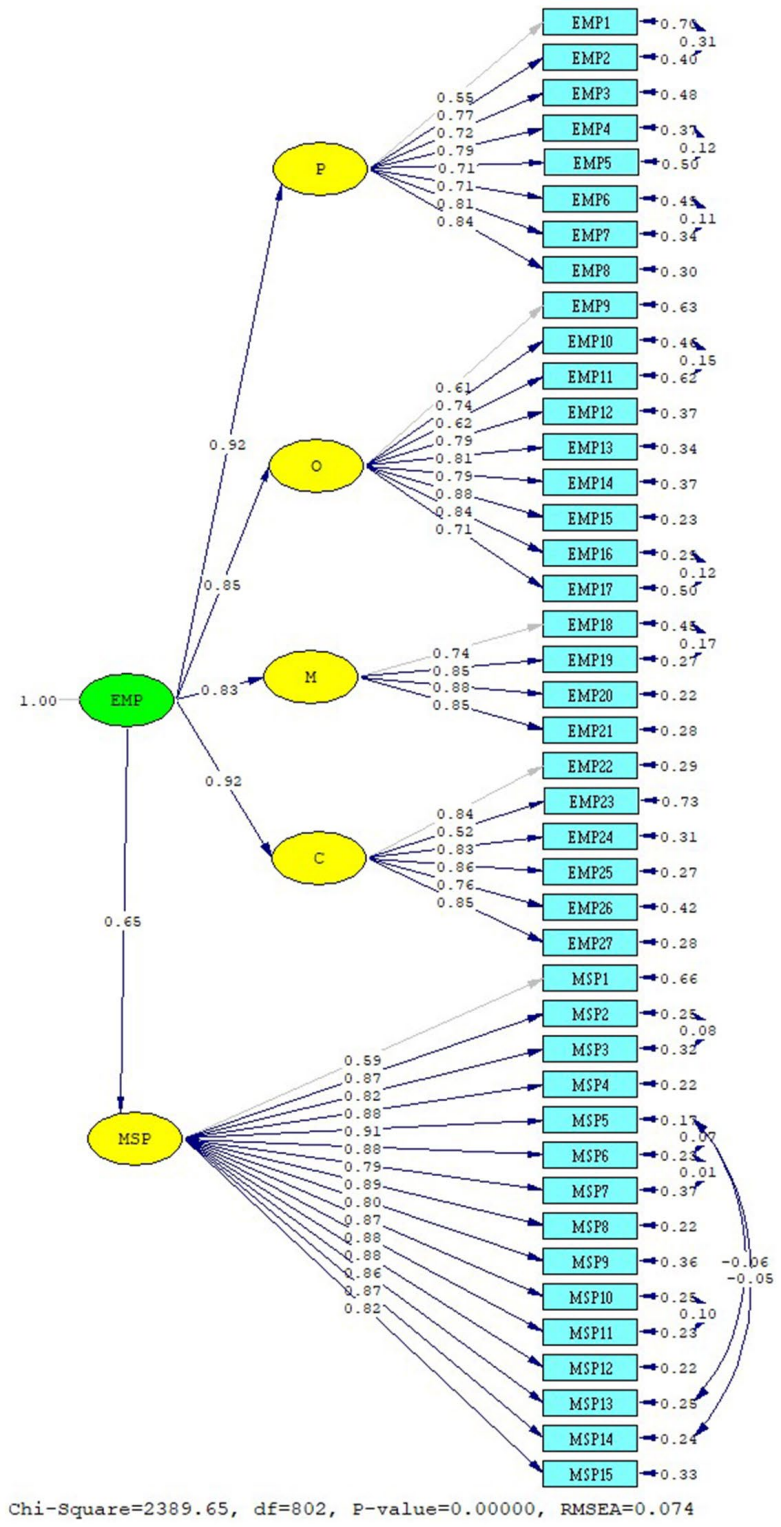
EMP and MSP scale standardized solution graph.

**Figure 5:**
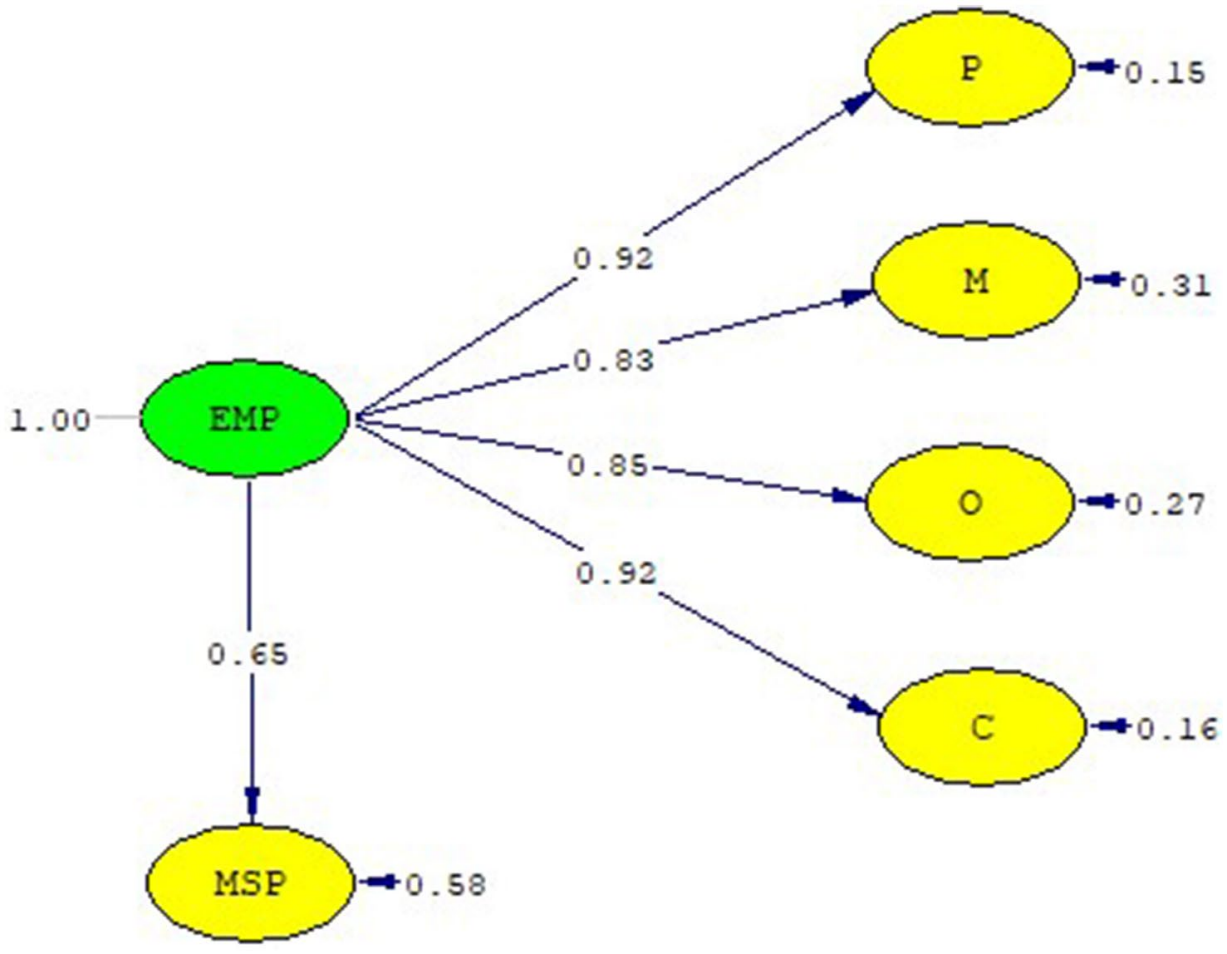
EMP and MSP scale structural model.

In the model, EMP Scale factors are defined as P, O, M, C, and MSP Scale as MSP. EMP Scale factors (P, O, M, C) are treated as an independent variable (external) and an MSP-dependent (internal) variable. In the model, P: EMP1-8, O: EMP9-17, M: EMP18-21, C: EMP22-27, and MSP Scale items: MSP1-MSP15 are measured. Arrows show the relationship between variables. The relationship between the EMP Scale and MSP Scale was analyzed. The analysis was performed using the maximum-likelihood method in Lisrel Estimated 8.8. The normality tests were examined for kurtosis, skewness values, and multicorrelation. Examining the fit of the model with the data, it was determined that the factor load was higher than 0.40, error variances were lower, and *t*-values were > 1.96 (63, 64). SEM path coefficient *t*-values were found to be between 8.77 and 13.3 (Table 7). In the evaluation of the SEM analysis, the fit between the model and theory is decided according to the fit index values available in the literature. Various fit values are used in the evaluation phase of the research model and the data. During the evaluation of the data with the research model, various fit values were used. Even if the factor loadings of the model are good, the decision is made according to the success of the fit indices. By model: Chi-square/SD (CMIN/DF): 2.97, RMSE: 0.074, CFI: 0.98, sRMR: 0.048, NFI: 0.96, RFI: 0.0562, IFI: 0.98, RFI: 0.98, GFI: 0.76, AGFI: 0.73 (Critical N (CN) = 126.97) (Table 8). The fit values show that the data fit with the model. The standardized solution graph of the model was given in Figure 4. The research structural model is given in Figure 5. The item statistics regarding the SEM findings of the EMP and MSP Scales are given in Table 7.

**Table 7:** Model: EMP and MSP scales SEM results of study measures.

Faktör madde	Λ (madde yükü)	*R* ^2^	Hata varyansı	*t*	*p*	AVE	CR
						0.596	0.975
EMP1	0.55	0.31	0.66	12.98	0.051		
EMP2	0.77	0.61	0.38	11.86	0.032		
EMP3	0.72	0.52	0.57	12.34	0.046		
EMP4	0.79	0.62	0.41	11.68	0.035		
EMP5	0.71	0.49	0.42	12.39	0.034		
EMP6	0.72	0.50	0.44	12.29	0.036		
EMP7	0.81	0.67	0.32	11.29	0.029		
EMP8	0.84	0.69	0.30	11.07	0.027		
EMP9	0.61	0.37	0.49	12.97	0.038		
EMP10	0.74	0.54	0.49	12.43	0.039		
EMP11	0.62	0.38	0.72	12.94	0.056		
EMP12	0.79	0.63	0.40	12.00	0.033		
EMP13	0.81	0.66	0.39	11.81	0.033		
EMP14	0.79	0.63	0.39	12.01	0.032		
EMP15	0.88	0.77	0.24	10.51	0.022		
EMP16	0.85	0.71	0.28	11.26	0.025		
EMP17	0.71	0.50	0.56	12.50	0.045		
EMP18	0.75	0.55	0.49	11.58	0.042		
EMP19	0.85	0.72	0.30	9.89	0.030		
EMP20	0.88	0.78	0.22	8.77	0.026		
EMP21	0.85	0.72	0.29	10.09	0.029		
EMP22	0.84	0.71	0.27	11.21	0.024		
EMP23	0.52	0.27	0.71	13.15	0.054		
EMP24	0.83	0.69	0.29	11.42	0.026		
EMP25	0.86	0.73	0.29	10.96	0.026		
EMP26	0.76	0.58	0.39	12.22	0.032		
EMP27	0.85	0.72	0.27	11.11	0.024		
						0.71	0.97
MSP1	0.59	0.34	0.71	13.33	0.054		
MSP2	0.87	0.75	0.34	12.52	0.027		
MSP3	0.82	0.68	0.50	12.80	0.039		
MSP4	0.88	0.78	0.30	12.38	0.024		
MSP5	0.91	0.83	0.25	11.35	0.022		
MSP6	0.88	0.77	0.33	12.20	0.027		
MSP7	0.79	0.62	0.53	12.93	0.040		
MSP8	0.89	0.78	0.30	12.34	0.024		
MSP9	0.80	0.63	0.49	12.94	0.037		
MSP10	0.87	0.75	0.37	12.53	0.030		
MSP11	0.88	0.77	0.34	12.41	0.027		
MSP12	0.88	0.78	0.34	12.38	0.028		
MSP13	0.86	0.75	0.40	12.34	0.032		
MSP14	0.87	0.76	0.34	12.29	0.028		
MSP15	0.82	0.67	0.57	12.87	0.045		

*P* < 0.05* AVE > 0.50, CR > 0.70, R^2^: coefficient of determination.

**Table 8:** Model EMP and MSP fit values.

Model fit value											
*χ*^2^/df	*p*	RMSEA	CFI	GFI	AGFI	NNFI	NFI	IFI	sRMR	RMR	RFI
2.97	0.000	0.074	0.98	0.76	0.73	0.97	0.96	0.98	0.048	0.056	0.98

Critical N (CN) = 126.97.

In the study, it was seen that the model of the relationship between variables was supported by the variance–covariance matrix. A high positive relationship was found between epidemic management and manager support (0.66). The relationship between the variables that make up the research model is presented in Table 9. In the relationship between the independent variables and the dependent variables, a positive relationship was found between the perception of manager support and the sub-dimensions of epidemic management. A positive relationship was found between manager support and the sub-dimensions of epidemic management, namely planning, organization, management, and control. As a result of the study, the relationship between EMP and MSP was tested using both Pearson correlation analysis and SEM.

**Table 9:** Covariance matrix of latex variable.

Variable						
	MSP	P	O	M	C	EMP
MSP	1.00					
P	0.61	1.00				
O	0.54	0.81	1.00			
M	0.53	0.76	0.74	1.00		
C	0.66	0.85	0.78	0.80	1.00	
EMP	0.66	0.91	0.86	0.83	0.91	1.00

MSP, Manager Support Perception; EMP, Epidemic Management Perception; P, Planning; O, Organization; M, Management; C, Control.

As a result of the research, the perception of epidemic management and manager support is related. The research hypothesis H1: “There is significant relationship between healthcare workers’ perception of epidemic management and manager support during the COVID-19 pandemic” was accepted.

## 5 Discussion

The COVID-19 pandemic has been a difficult process all over the world. The healthcare sector was the most affected by the COVID-19 pandemic. Identifying deficiencies in process management in health institutions during the epidemic process is important in order to be prepared for future epidemics. Scale development studies on crisis management are more prominent in the literature (37–42) (Supplementary Table S1). We believe that the scale development study conducted in the research to determine the practices that can guide health institutions in preparing for the pandemic and managing the process has made a significant contribution to the literature. The scale was developed and added to the literature by determining the planning, organization, management, and control activities in epidemic management based on the perception of healthcare professionals. The epidemic management in healthcare institutions through the perception of healthcare workers and emphasizing. The importance of manager support in this process. In studies on epidemic management, Liang (8) stated that isolation area practices are among the priority issues in order to prevent COVID-19 infection and spread during the epidemic process. Taylor et al. (65) emphasized the importance of determining material, equipment and facility capacity, ensuring employee safety, legal process and establishing systems against the epidemic, such as infection control and occupational safety practices. In addition, Aminizadeh et al. (66) emphasized that the existence of standards or guidelines should be seen as the key to success in order for the epidemic process to function properly. In studies evaluating the situation of healthcare workers who played an active role in the epidemic process, it has been observed that people who have contracted COVID-19 develop traumas resulting from physical and psychological stress. Piras et al. (67) revealed four main themes emerged: “emotion of fear; isolation and loneliness; unawareness about the gravity of the situation as a protective factor; “Long COVID” as consequences of the disease on physical and psychological health.” Boyacı and Söyük (44) stated that the psychological impact of the epidemic on healthcare workers is high, and the perception of control is low. Dehnavieh and Kalavani (17) offered suggestions to healthcare managers during the epidemic process. For healthcare managers, the importance of leadership and motivation in epidemic management, recognition and approval of employees, work and life balance of employees, support from colleagues, and organization of working and rest hours was emphasized. A positive perspective toward the organization and its manager will lead to an increase in the employee’s motivation, performance, and service quality. In this case, it will ensure the development of the organization (68). The studies in the literature on the COVID-19 outbreak focus on only one dimension. In our study, unlike other studies, pandemic management was evaluated as a whole. It was not only the outbreak preparation phase but also the organization, management, and control dimensions that were addressed, and the process was defined from the perception of healthcare professionals from beginning to end. For this reason, the research is different.

Healthcare workers are the key to the success of management practices in healthcare institutions. One of the most important issues that healthcare professionals care about when evaluating epidemic management practices during the pandemic is the support they receive from their managers.

A positive and supportive relationship between employees and managers is necessary for the easy recovery of the outbreak process. Supporting employees, protecting employees against the stress and anxiety caused by the epidemic, and ensuring employees’ participation in decisions will increase trust in managers (21). On the contrary, employees who do not receive sufficient support and value from their managers will tend to distance themselves from the work environment (69). Scholars have suggested that supervisor support plays a role in developing positive attitudes among employees toward their organizations (23). Research indicates that manager support plays an important role in keeping employees’ psychology and wellbeing high during crisis/epidemic processes (70). Babin and Boles (71) stated that employees’ perceptions of manager support are inversely proportional to job stress and directly proportional to job satisfaction. Um-e-Rubbab et al. (49) found that manager support had a positive psychological and physical impact on employees during the COVID-19 outbreak. In a study conducted in South Korea, Kim et al. (72) examined the impact of managerial support and communication during the epidemic process and found that managerial support was a key factor in enhancing employees’ resilience, coping abilities, and effectiveness. The study emphasized that managers should demonstrate guiding behaviors by understanding the stress caused by the outbreak and take necessary steps to protect their employees. It is also highlighted that institutions need to develop effective policies to combat the epidemic (72). In their 2023 study, Taşkıran (37) reported that nurses did not feel professionally secure during the pandemic due to increased stress, disruptions in working conditions, and a lack of managerial support. The study recommends that nurse managers take initiatives to enhance nurses’ sense of security by organizing training programs, providing support, and fostering collaboration.

Supervisors should know the issues and problems that employees face and should be able to empathize with their employees. For this, supervisors need to be trained and informed. It is observed that employees who receive support from their managers during the COVID-19 process have reduced anxiety and stress (49). According to the findings of our research, managerial support plays an important role in the adoption of epidemic management implemented in health institutions by employees. The existence of a high level of relationship between epidemic management and manager support, employees’ positive attitudes and behaviors toward epidemic management strongly indicates the importance of manager support. These studies support our research. Kottke and Sharafinski (73) found in their research that in businesses with low managerial and organizational support, employees’ job stress and depression levels were high, and organizational commitment and job satisfaction were low. It has been determined that in the conditions of uncertainty that arise in the workplace, employees who receive the necessary information and support from their managers have an effect in reducing the negative impact of the crisis. Managers support employees by providing information that will ease their concerns during times of crisis. It is thought that the negative consequences of the COVID-19 pandemic on healthcare workers can be mitigated with managerial support. Charoensukmongkol and Phungsoonthorn (74) suggested in their study that the degree of perceived uncertainty and emotional exhaustion caused by the COVID-19 crisis among employees can be alleviated with managerial support. Our research shows that healthcare professionals’ positive reception and adoption of epidemic management practices in healthcare institutions depends on the strength of managerial support. These studies support our research. Managerial support can increase resilience and effectiveness in employees who are experiencing uncertainty and emotional tension during the pandemic. Managers play an important role in helping employees overcome threats and fears during the pandemic (72).

Supporting employees to adapt to changes that occur during the COVID-19 pandemic (e.g., staggered shifts, remote work, and workload) provides psychological relief (75). Our research found that the most important factor in healthcare professionals’ positive perceptions of epidemic management was the support they received from their managers.

### 5.1 Limitation

This study has some limitations. First, the study was conducted in a single healthcare institution; therefore, the generalizability of the results to all healthcare institutions and healthcare workers may not be guaranteed. It would be advantageous to replicate and expand this study using various samples to increase the applicability of the findings. Longitudinal studies are needed to support the generalizability of the findings. Further investigation of the effects in other settings (e.g., private or public) is needed. Second, the study was designed as a cross-sectional descriptive study, and the cross-sectional design of the study only provides a snapshot of the relationship between the variables. The use of the cross-sectional design poses significant threats to the validity and generalizability of the study. To address this limitation, future studies could expand the sample to include all healthcare institutions, even more diverse regions internationally, to increase the external validity of the results. The study group consists of healthcare workers. The study is limited to healthcare workers. Since the time and cost elements of the study are taken into consideration, it is limited to healthcare workers (physicians, nurses, other healthcare workers) in different positions working in a university hospital in Istanbul. Participants were assured that their answers would remain anonymous and confidential. Nevertheless, it continues to be difficult to eliminate institutional concerns during the survey response process. The answers given to the survey in the study are considered correct. Data will not be collected from people other than healthcare workers, and incomplete or incorrect information will not be included in the research.

### 5.2 Strengths of the research

This study demonstrates several methodological strengths. In the process of developing the scale, face validity, content validity, CFA, and construct validity procedures were rigorously applied. For reliability, the Cronbach’s alpha coefficient and the significance of the difference between the upper and lower 27% groups were used to establish the scale’s reliability and validity. EFA and CFA were conducted two times using different datasets with the same sample characteristics, which further reinforced the construct validity. The research also contributes to the literature by introducing a unique scale that evaluates epidemic management practices in healthcare institutions based on employee perceptions—a gap previously unaddressed. Additionally, the relationship between epidemic management and managerial support was analyzed using SEM through the LISREL 8.8 program. The scarcity of studies that model this relationship in the literature further underscores the strength and originality of the research.

## 6 Conclusion

In the research, an “EMP” Scale development study was conducted to determine how healthcare professionals perceive epidemic management practices in healthcare institutions. As a result of validity and reliability analyses, the EMP Scale was introduced to the literature as a scale with high reliability and validity. The EMP Scale was developed as a scale based on a single-center hospital. Further testing of the scale in different institutions and regions is required to check its validity.

A model was created using SEM to determine the relationship between healthcare workers’ perceptions of epidemic management and manager support during the pandemic process. The theoretical model was modeled and validated as a whole. When the relationship between the model variables was examined, a high positive and significant relationship was found between healthcare workers’ perceptions of manager support and epidemic management. A high-level, positive, significant relationship was found between manager support and epidemic management sub-dimensions. There is no study in the literature showing a high-level relationship between epidemic management and manager support. Therefore, it is thought that the study makes a significant contribution to the literature.

### 6.1 Suggestions

Recommendations for healthcare managers and institutions as a result of the research:

- The EMP Scale should be tested further in other healthcare facilities and in different settings to check its validity.
- Preparation of plans and strategies for the epidemic/disaster/crisis process, ensuring periodic control and supervision of the process.
- During the pandemic, attention should be paid to issues such as shortage of protective equipment and personnel, regulation of physical conditions, overtime, shift work, and provision of physical and psychological support to employees.
- Regular studies should be carried out to adopt a supportive management approach in the institution.
- Informing health managers about the importance of managerial support and organizing necessary training activities.
- Developing methods to regularly collect employee opinions in order to evaluate manager performance and competencies.
- It is recommended that the scale be translated into other languages and cross-cultural validation studies conducted.
- It is recommended that the findings obtained as a result of the research be used in similar studies and that the developed scale be adapted and used in other institutions and organizations.

## Data Availability

The raw data supporting the conclusions of this article will be made available by the authors, without undue reservation.

## Author’s note

This article is derived from Hafize Boyacı’s doctoral thesis titled “The relationships between the perception of epidemic management and manager support and work stress, workload and work performance: Scale development and adaptation study’’.
