# Supplementary material for: The relationship between healthcare workers’ perceptions of epidemic management and manager support in a healthcare institution during the COVID-19 pandemic: scale development study
Source: Front Public Health. 2025 Jul 15;13:1477961. doi: 10.3389/fpubh.2025.1477961 (PMC12304002; doi:10.3389/fpubh.2025.1477961)
Supplement: Supplementary file 1 [file Table_1.docx]

Supplementary Material

***Supplementary Material***

**S1: Scales related to epidemic/crisis management.**

| **Researchers** | **Scales** | **ResearchVariables/Subject** | **Research Sample** |
| --- | --- | --- | --- |
| Nevala, 2021 | Semi-structured literature review and semi-formal thematic analysis on COVID-19 | The output was five themes: capacity building, staff management, infection prevention, emerging issues and general experiences. | Nurse managers of four COVID-19 care centres in the Helsinki region |
| Sayın, 2008 | ‘‘Crisis Management Scale’’ “Crisis Management Scale” was used in the theses of Cin Z., 2021, (9 hospitals 36 managers) Atabay B. 2019, Koyuncu S. 2019 adapted the scale to healthcare professionals in their theses. | Crisis management scale: 3 sub-dimensions: “Before the Crisis, When the Crisis Occurs and After the Crisis Occurs”. | School administrators working in secondary schools in Istanbul n= 603 |
| Aksu and Deveci, 2009 | “Crisis Management Scale”. “Crisis Management Scale” was used in the theses of Doğan S. 2021 and Balaban S. 2021. | “It consists of three sub-dimensions: "Pre-Crisis Period", "Crisis Period", "Post-Crisis Period". 2006-2007 | Teachers working in primary schools in Izmir/Buca district n= 200 |
| Pensore, 2000 | “The Role of Perception in Crisis Planning” scale development. | The impact of crisis perception on crisis management activities and threats and opportunities when planning crisis management activities. |  |
| Taşkıran, 2021 | It is based on the article titled “The Role of Perception in Crisis Planning” published by J.M. Pensore in Public Relations Review v26 no2 p.155-171 in 2000 and the research he conducted within that research. | The 32-item scale form taken from the study was translated into Turkish by Prof. Dr. Çisil Sohodol. | 128 corporate communication and public relations managers from 240 medium and large-scale institutions operating in Turkey |
| Çoban and Özel, 2020 | Semi-structured interview questions created by the researcher | Crisis management practices in hotel management during the Covid Pandemic. | Manager of 13 hotel businesses in Eskişehir province |
| Louhela, 2020 | Comparison between the time before and after the coronavirus situation and the activities during the quarantine. | To compare 16 UK and 53 Finnish civil society organisations and understand how they are coping with the coronavirus crisis | (n=79) |
| Rajamäki, Vilén, and Waltari, 2021 | A questionnaire consisting of 44 statements for nursing staff created based on literature review. |  | Data (n= 33) were collected using medical databases and literature at an outpatient clinic from southwestern Finland. |
| Zhang, M. et al. 2020 | A short test consisting of eight items assessing knowledge of epidemic infections. | To determine the situation and feelings of healthcare workers regarding the epidemic. | n= 1357 in 1825 general hospitals in Henan Province, China |
| Mishraa A. et all.2021 | A healthy health management model | • Public health management; • Patient care management; • Health technology management categories. Effectiveness in health services is necessary to make society healthier. | N=424 health workers |
| Cowden et al. 2010, | Designing a scale to determine the relationship between health worker knowledge, opinions, concerns, professionalism, behavioral intention, barriers and motivators, personal and professional preparation | Characteristics of workers who reported being willing to work during the pandemic; importance of barriers and motivators versus those who were reluctant or unsure. | Healthcare workers at The Children's Hospital in Denver, Colorado n=778 |
| Dehnavieh and Kalavani, 2020 | Recommendations presented to management during healthcare use | There are 9 suggestions for health managers to determine a scientific framework for epidemic management. | Literartür review |
| Boyacı and Söyük, 2021 | “COVID-19 Psychological Impact Scale” | To reveal the psychological impact of the COVID-19 pandemic on | laboratory workers  n=82 |
| Chatzittofis. et al. 2021 | Demographic information was collected using a 7-item questionnaire assessing POS, the “Patient Health Questionnaire,” the “Impact of Events Scale-Revised” measuring symptoms of post-traumatic stress disorder (PTSD), and the “Perceived Stress Scale” assessing perceived stress. | The link between the COVID-19 pandemic and mental distress in healthcare workers (HCWs) | Data collected on 424 healthcare workers during the first wave of the pandemic |
| Azadian SH, Shirali GHA, Saki A. 2014 | Designed a survey to evaluate crisis management based on the Resilience Engineering approach | Resilience Engineering (RE) practices to increase the efficiency of crisis management. | Iran in the year 2013, and 113 nurses |
| Harris GH, Rak KJ. 2021 | The 2017–2018 seasonal influenza outbreak in the United States, with its higher disease severity, wider geographic spread, and longer duration compared to previous seasons, has highlighted the increasing strain on acute care hospital systems. | Semi-structured telephone interviews were conducted with capacity management staff in short-term acute care hospitals in the United States.  . | A sample size was not determined at the beginning of the study, but based on previous experience, approximately 30-40 interviews were required to reach thematic saturation. Thematic saturation was achieved after 53 interviews.. |
| Liu Y. et al. 2020 |  | Comprehensive study of emergency management of nursing human resources and supplies of a large general hospital during the COVID-19 outbreak | It listed effective management measures to respond to the epidemic. |
| Steier J, Moxham J., 2020 | A load-capacity model for healthcare delivery was created to explain the factors that need to be considered during the pandemic and how saturation of the system can be prevented, and to investigate what the implications are for long-term investment decisions in healthcare. | İllustrates the resources that should be considered when addressing demand in a pandemic. | Qualitative reserach |
| Bailey K, Breslin D., 2021 | Over the past 20 years, the International Journal of Management Reviews (IJMR) has published many research reviews that bring together key findings from major bodies of research relevant to understanding how organizations can address such major challenge | **COVID-19 Pandemic: What can we learn from past research on organizations and management?** | Reflecting on this work, the journal has selected a number of new reviews that are relevant to both practitioners and academics in the current crisis. |
| Pfarrer JB, MD. Short CE, Coombs WT., 2017 | The Academy of Management Review article laid the foundation for subsequent developments in the literature; therefore, their article was examined*.* | The model identifies crisis management stages while synthesizing commonalities across perspectives and offering directions for future research. |  |
| Bernardino E, Nascimento JD, Raboni SM, Sousa SM., 2021 | Care management strategies are structured on the basis of service dynamics; physical structure; human resources; professional and user safety. Final assessments: measures to prepare for a pandemic, change infrastructure and processes, manage employees and users, infection prevention strategies and clinical recommendations. These measures are the necessary methods to improve the quality of care provided to users with COVID-19 and reduce the risk of viral transmission to other users or healthcare professionals. | To report the experience of implementing care management strategies in coping with the COVID-19 pandemic in a teaching hospital. | It is a report of the experience of health managers working in the largest public hospital in Paraná. In order to develop care management strategies to cope with the COVID-19 pandemic, national and international recommendations to cope with COVID 19, such as WHO regulations, the Ministry of Health, the Brazilian Health Surveillance Agency (Agência Nacional de Vigilância Sanitária), the Ministry of Health of Paraná and the Ministry of Health of the Municipality of Curitiba, and past management expertise were used |
| Porter TH, Rathert C, Ayad S, Messina N., 2021 |  | The importance of building an organizational-level team (i.e., an organization-wide team) has been examined as the key to successfully mitigating organizational crises. | Interview with Dr. Mark Taylor, Chief Surgical Operations Officer at Cleveland Clinic, who recounts her first-hand experiences during the pandemic and is a key architect of Cleveland Clinic’s COVID-19 response |
| Lotfollahzadeh A, Moslehı S, Ebrahımı H., 2019 | Crisis Management Performance Assessment survey | Scale development to have a powerful tool to measure the performance of crisis management in the sector |  |
| Barroa K, Malone A, Mokedea A, Chevancec C., 2020 | It details the measures taken in public institutions to cope with the COVID-19 outbreak. | The first strategy, the organizational evolution towards “all COVID,” the coordination between various stakeholders, and the strategy to ensure continuity of care are detailed. |  |

**Referans:**12,14, 15,16, 17, 26,27,28, 29,30,31,32,33,34,35,36,37,38,39,40,41,42,**43,44**
