# Supplementary material for: The relationship between healthcare workers’ perceptions of epidemic management and manager support in a healthcare institution during the COVID-19 pandemic: scale development study
Source: Front Public Health. 2025 Jul 15;13:1477961. doi: 10.3389/fpubh.2025.1477961 (PMC12304002; doi:10.3389/fpubh.2025.1477961)
Supplement: Supplementary file 2 [file Table_2.docx]

Supplementary Material

***Supplementary Material***

**S2 Epidemic Management Perception Scale**

|  | **EPIDEMIC MANAGEMENT PERCEPTION SCALE** |
| --- | --- |
| **FACTOR** | **ITEMS** |
|  | **In the institution you work in;** |
| **Planning** | **1** Existing plans are sufficient for employees to maintain job continuity |
|  | **2** Existing plans for epidemic management are adequately implemented |
|  | **3** All employees are aware of the epidemic management plan |
|  | **4** Training programs are organized for employees on what to do in case of an epidemic/crisis. |
|  | **5** There are inventories in the institution's archives that contain the knowledge and experiences of employees regarding the pandemic process. |
|  | **6** Support is provided for the emergency service to be performed correctly, quickly and on time. |
|  | **7** Security rules and procedures are applied |
|  | **8** Employees are informed about all announcements regarding the epidemic in a timely manner. |
| **Organization** | **9** There are staff backup plans to ensure service continuity in epidemic/crisis situations. |
|  | **10** Necessary arrangements are made for the increased workload. |
|  | **11** Personnel are transferred to the required units to reduce the increasing workload |
|  | **12** Physical conditions are provided for employees to work comfortably. |
|  | **13** Necessary support is provided to protect employees from the physical and psychological impact of the pandemic. |
|  | **14** Employees participate in decision-making regarding epidemic management |
|  | **15** Provides solutions to problems and complaints faced by employees during the pandemic. |
|  | **16** Activities that will increase employee motivation are implemented. |
|  | **17** A reward mechanism is implemented for employees' performance during the pandemic |
| **Management** | **18** All units and teams act with a common perspective to overcome the epidemic process with the least damage. |
|  | **19** Collaborations and communication channels are used to make correct decisions.. |
|  | **20** Correct communication channels are used throughout the epidemic process. |
|  | **21** Effective communication is established with managers for the implementation of procedures. |
| **Control** | **22** Managers are in contact with other hospitals, national and international organizations |
|  | **23** Collaboration between employees and managers ensures that the process is easy to overcome. |
|  | **24** The institution's outbreak management practices are successful |
|  | **25** Managers' responsibilities, roles and objectives in epidemic management are clearly defined. |
|  | **26** The institution has the ability to collaborate with people who are experts in epidemic management. |
|  | **27** Institution managers use the necessary software and hardware systems to organize actions during the epidemic. |
